# Deregulation of lipid metabolism pathway genes in nasopharyngeal carcinoma cells

**DOI:** 10.3892/mmr.2012.1253

**Published:** 2012-12-28

**Authors:** MAELINDA DAKER, SAATHEEYAVAANE BHUVANENDRAN, MUNIRAH AHMAD, KENZO TAKADA, ALAN SOO-BENG KHOO

**Affiliations:** 1 Molecular Pathology Unit, Cancer Research Centre, Institute for Medical Research, Kuala Lumpur 50588, Malaysia; 2 Department of Tumour Virology, Institute for Genetic Medicine, Hokkaido University, Sapporo 060-0815, Japan

**Keywords:** Epstein-Barr virus-encoded RNAs, fatty acid synthase, lipid metabolism, low-density lipoprotein receptor, nasopharyngeal carcinoma, quercetin

## Abstract

Nasopharyngeal carcinoma (NPC) is a unique tumour of epithelial origin with a distinct geographical distribution, closely associated with the Epstein-Barr virus (EBV). EBV-encoded RNAs (EBERs) are small non-polyadenylated RNAs that are abundantly expressed in latent EBV-infected NPC cells. To study the role of EBERs in NPC, we established stable expression of EBERs in HK1, an EBV-negative NPC cell line. Cells expressing EBERs consistently exhibited an increased growth rate. However, EBERs did not confer resistance towards cisplatin-induced apoptosis or promote migration or invasion ability in the cells tested. Using microarray gene expression profiling, we identified potential candidate genes that were deregulated in NPC cells expressing EBERs. Gene Ontology analysis of the data set revealed that EBERs upregulate the cellular lipid metabolic process. Upregulation of low-density lipoprotein receptor (LDLR) and fatty acid synthase (FASN) was observed in EBER-expressing cells. NPC cells exhibited LDL-dependent cell proliferation. In addition, a polyphenolic flavonoid compound, quercetin, known to inhibit FASN, was found to inhibit proliferation of NPC cells.

## Introduction

Nasopharyngeal carcinoma (NPC) is a cancer with distinct ethnic and geographic distribution. The cancer is common in Southern China, North Africa and South East Asia, including Malaysia (1). Approximately 95% of NPC cases (2) are associated with latent infection of the Epstein-Barr virus (EBV), a γherpesvirus of the *Lymphocryptovirus* genus. EBV-infected NPC cells exhibit type II latency and express EBV-encoded RNAs (EBERs; small, non-coding, nonpolyadenylated), EBV-associated nuclear antigen-1 (EBNA1), latent membrane proteins 1 and 2 (LMP1, 2A and 2B), *Bam*H1 A RNA transcripts (BART) and BARF1 protein. LMP1, BARF1 and EBNA1 are oncogenic (1).

EBER-1 and EBER-2 are highly transcribed nuclear RNAs, composed of 167 and 172 nucleotides, respectively (3). Their abundance in infected cells has led to the use of *in situ* hybridisation (ISH) for the detection of EBV infection in tissue specimens (3). Numerous studies have analysed the role of EBERs in Burkitts’ lymphoma (4–7). In addition, a small number of reports have described the role of EBERs in epithelial malignancies. These include reports that EBERs confer resistance to pIC-induced apoptosis (8) and induce insulin-like growth factor 1 (IGF-1) (9,10).

To understand the role of EBERs in NPC, we established stable expression of EBERs in HK1, an EBV-negative NPC cell line, by transfecting HK1 with an expression vector containing 10 tandem repeats of EBER-1 and-2 (4). Our findings indicated that EBERs enhanced cell proliferation. Gene expression profiling by microarray revealed that members of the cellular lipid metabolic process were aberrantly expressed. We found that EBER-positive cells demonstrated the upregulation of low-density lipoprotein receptor (LDLR) and fatty acid synthase (FASN). NPC cells were identified to undergo low-density lipoprotein (LDL)-dependent cell proliferation and were sensitive to a polyphenolic compound, quercetin.

## Materials and methods

### Cell lines and culture

HK1, EBV-negative (11) and C666-1, EBV-positive NPC cell lines (12) and all stable cell lines established from HK1 thereafter, were maintained in RPMI-1640 medium supplemented with 10% heat-inactivated foetal calf serum (FCS), 10 U/ml of penicillin and 10 μg/ml streptomycin (all obtained from Gibco-BRL, Carlsbad, CA, USA) and cultured at 37˚C in a 5% CO_2_ humidified atmosphere. The identity of HK1 and C666-1 were validated by DNA fingerprinting using the AmpFISTR Identifiler^®^ polymerase chain reaction (PCR) amplification kit (Applied Biosystems, Foster City, CA, USA). The short tandem repeat profiles were consistent with published data (13). Tests for *Mycoplasma* using *e*-myco™ *Mycoplasma* PCR Detection kit (Intron Biotechnology Co. Ltd., Gyeonggi-do, Korea) were conducted routinely and contamination-free cells were used throughout the study.

### Plasmids and transfection

The EBER plasmid (designated as pcDNA 3 Eks10) contained 10 tandem repeats of the EBER-1 and -2 subfragments inserted into a pcDNA 3 vector (4). Empty vector pcDNA 3.1 was used as a control. Plasmids were transfected into HK1 cells using Lipofectamine 2000 (Invitrogen Life Technologies, Carlsbad, CA, USA), according to the manufacturer’s instructions. Stable transfectants were selected by culturing the cells in medium supplemented with 10% FCS containing G418 (Sigma-Aldrich, St. Louis, MO, USA) for 6 days. Resistant colonies were pooled and used for subsequent experiments.

### Confirmation of EBER expression by reverse transcription (RT)-PCR

Total RNA was extracted from each cell line using TRIzol^®^ reagent (Invitrogen). DNase I (Promega Corporation, Madison, WI, USA) treatment was performed according to the manufacturer’s instructions. cDNA was synthesized from 2 μg total RNA using the High Capacity cDNA Reverse Transcription kit (Applied Biosystems) followed by PCR amplification, using DyNAzyme™ II PCR Master Mix (Finnzymes, Espoo, Finland) in a thermal cycler. Primer sequences were as follows: EBER-1 forward, 5′-AGGACCTACGCTGCCCTAGA-3′; reverse, 5′-AAAACATGCGGACCACCAGC-3′; EBER-2 forward, 5′-CAACGCTCAGTGCGGTGCTA-3′; reverse, 5′-CAGCGGACAAGCCGAATACC-3′. β-actin (ACTB), a housekeeping gene, was amplified as the internal control using the primers: ACTB forward, 5′-TCATCACCATTGGCAATGAG-3′ and reverse, 5′-CACTGTGTTGGCGTACAGGT-3′. PCR products were separated on 2% agarose gels and visualized by ethidium bromide staining.

### MTS assay and light microscopy

Cells were seeded at 5,000 cells/well into 96-well microtiter plates in 100 μl culture medium supplemented with 1 or 10% FCS for 5 days at 37˚C in a 5% CO_2_ atmosphere. The number of viable cells at each time point was measured using the CellTiter 96^®^ AQ_ueous_ One Solution Cell Proliferation (MTS) assay (Promega Corporation), according to the manufacturer’s instructions. Absorbance at 490 nm was read using the EnVision Multilabel Plate Reader (Perkin-Elmer, Waltham, MA, USA) and subtracted with non-specific absorbance measured at 630 nm. Wells containing the appropriate medium but without cells served as the blank. Population growth was calculated as % of viable vs. cells on Day 0 (arbitrarily assigned viability = 100%). Experiments were performed in triplicate. Results were expressed as the mean ± SEM.

To observe the effects of EBERs on the cells by light microscopy, 1.8×10^5^ cells/well were seeded into 6-well plates in 2.5 ml culture medium supplemented with 1 or 10% FCS for 2 days at 37˚C in a 5% CO_2_ atmosphere. Cell morphology was observed under a Leica DM IRB (Leica Microsystems, Wetzlar, Germany) inverted microscope equipped with HC PLAN S 10x/22 ocular lens and PH1 N PLAN 10x/0.25 objective lens.

To determine the effects of human LDL supplementation, 96-well microtiter plates were seeded with 5,000 cells/well in 100 μl serum-free culture medium only or added with 50–100 μg/ml LDL (Sigma-Aldrich) for 6 days at 37˚C in a 5% CO_2_ atmosphere. The MTS assay was performed as described above. For blockade of receptor-ligand interaction, 5 μg/ml goat anti-human LDLR antibody (R&D Systems, Minneapolis, MN, USA) was added to the cells and incubated for 24 h. Following this, culture medium was aspirated and replaced with serum-free culture medium supplemented with LDL or serum-free culture medium only and cultured for 6 days, following which the MTS assay was performed. Population growth was calculated as % of viable vs. control cells (arbitrarily assigned viability = 100%). Experiments were performed in triplicate. Results were expressed as the mean ± SEM.

### Apoptosis analysis assay

HK1 cells (1.2×10^6^) were seeded in 10-cm culture dishes and were allowed to adhere overnight. Following this, cells were treated with 50–100 μg/ml cisplatin or left untreated (as control). Culture dishes were re-incubated for an additional 24 h. Apoptosis was determined using a FACSCalibur flow cytometer (BD Biosciences, San Jose, CA, USA) and the BD Pharmingen FITC Annexin V apoptosis detection kit (San Diego, CA, USA), according to the manufacturer’s instructions.

### Scratch-wound assay

A 6-well plate was seeded with 1×10^6^ cells/well and incubated overnight until attachment and confluence were achieved, followed by treatment with 3 μg/ml Mitomycin C (Sigma-Aldrich) for 2 h. Three wells were seeded per stable cell line. A scratch mimicking a wound was made using a 200 μl pipette tip. Wells were washed thoroughly with PBS to remove cells detached by scratching. Zero hour images were captured using a Leica DM IRB inverted microscope. The migration pattern was captured 24 h later. Using TScratch, a software tool to analyse wound healing assays developed by the Koumoutsakos group (CSE Lab), at ETH Zürich (14), open (area unoccupied by cells) and the closed image areas (area where cells have migrated), were calculated.

### Invasion assay

Cells were grown to near confluence overnight following which cell suspensions in serum-free medium were prepared. A total of 1.8×10^5^ cells/well were seeded in the apical chamber of BD Falcon FluoroBlok 24-Multiwell Insert System uncoated or coated with BD Matrigel Basement Membrane Matrix. Differential *in vitro* invasive properties of the cells were assessed using the BD BioCoat Tumor Invasion System (all obtained from BD Biosciences), according to the manufacturer’s instructions. Culture medium supplemented with 10% FCS was used as the chemoattractant in the basal chamber. Cells were labelled for quantification post-invasion with calcein AM fluorescent dye. Fluorescence readings were obtained using a bottom-reading fluorescent BioTek H4 (Winooski, VT, USA) plate reader.

### Microarray analysis

Quality of total RNA isolated from stable HK1 transfectants was determined using the RNA 6000 Nano kit in a 2100 Bioanalyzer (Agilent Technologies, Waldbronn, Germany). Total RNA was labelled using GeneChip^®^ Whole Transcript Sense Target Labelling assay (Affymetrix, Santa Clara, CA, USA) and then hybridized onto the GeneChip^®^ Human Gene 1.0 ST array (Affymetrix), in triplicate. Microarray scanning and data acquisition were performed using an Affymetrix 3000 7G scanner. Minimum Information About a Microarray Experiment compliant microarray data were deposited in the NCBI’s Gene Expression Omnibus (15) (http://www.ncbi.nlm.nih.gov/geo/; accession no. GSE 29123). Differential expression of candidate genes was identified using the unpaired t-test and GeneSpring GX 10 (Agilent Technologies). P<0.05 was considered to indicate a statistically significant difference and the threshold was set at ≥2-fold change. Results were categorised using the Gene Ontology (GO) database to identify involvement in biological processes, cell components and molecular functions.

### Quantitative real time PCR (qRT-PCR)

cDNA generated from total RNA (as described) was amplified using Power SYBR-Green Master Mix (Applied Biosystems) in the Applied Biosystems 7500 Fast Real-Time PCR system and analysed with 7500 software v.2.0.4. A series of diluted cDNA samples were used to generate standard curves to determine primer efficiency and melting curves to verify the presence of a single amplicon. Primer sets were as follows: sterol regulatory element binding protein (SREBF) 1 forward, 5′-AGTGACTCGGAGCCTGACA-3′; reverse, 5′-TATGGTAGACGCTGGTGGTATC-3′; SREBF2 forward, 5′-GCAGTGGTGGTAGTGGTAGCA-3′; reverse, 5′-GTGGGAACTGAGGTGGGAGAAA-3′; FASN forward, 5′-CAAAGAAGCCCATCTCCCG-3′; reverse, 5′-GCACCTCCTTGGCAAACAC-3′; LDLR forward, 5′-AGAAGAAGCCCAGTAGCGTGA-3′; reverse, 5′-TTGTGGCAAATGTGGACCTC-3′; peptidylprolyl isomerise A (PPIA) forward, 5′-GGCCAGGCTCGTGCCGTTTT-3′ and reverse, 5′-TGCTGTCTTTGGGACCTTGTCTGC-3′. ACTB and PPIA were consistently expressed at similar levels in HK1-vector and HK1-EBER. Therefore, these genes were used to normalize the expression levels of all other candidate genes. Experiments were performed in quadruplicates and control reactions were performed in parallel in the absence of reverse transcriptase or by substituting template cDNA with ultrapure water.

For RT-PCR of xenografts, snap frozen tumour was removed from liquid nitrogen and pulverized in 1 ml TRIzol^®^ reagent with a power homogenizer. The extracted total RNA was dissolved in DEPC-treated water and subjected to the procedure described for cell culture followed by PCR amplification to detect EBER-1 and -2. In addition, qRT-PCR was performed for *SREBF1* and *2*, *FASN* and *LDLR*.

### Western blot analysis

Cells (1.2×10^6^) were seeded in 10-cm plates and cultured in medium containing 10% FCS overnight in the absence or presence of 100 μM quercetin. Cells were lysed in 1X RIPA lysis buffer and boiled for 10 min. Quantity of protein in the cell lysate was determined by protein assay (Bio-Rad, Hercules, CA, USA). A total amount of 10 μg of protein was resolved in NuPAGE^®^ Novex^®^ Bis-Tris Mini Gels (Invitrogen) and electrotransferred to polyvinylidene fluoride membranes (Millipore, Bedford, MA, USA). Membranes were blocked using 5% skimmed milk and incubated overnight in primary antibodies diluted in 5% skimmed milk. Primary antibodies against FASN (Sigma-Aldrich), LDLR (Abcam, Cambridge, UK), β-actin (Santa Cruz Biotechnology, Inc., Santa Cruz, CA, USA) and monoclonal mouse anti-human Ki67 antigen (Dako Denmark A/S, Glostrup, Denmark) were utilised. The secondary antibody reaction was performed using anti-mouse or anti-rabbit horseradish peroxidase- conjugated IgG (Promega Corporation). Western Lighting^®^ Plus ECL substrate (Perkin Elmer) and autoradiography were used to visualize the protein expression. Densitometric analysis of X-ray films was performed on Alpha Imager System (ProteinSimple, Santa Clara, CA, USA) using Alpha View software.

### xCELLigence cell proliferation assay

Cells were seeded at a density of 5,000 cells/well into three E-Plate 16 (ACEA Biosciences, Inc., San Diego, CA, USA) containing 100 μl culture medium supplemented with 10% FCS/well. Following 48 h, 1 mM quercetin (C_15_H_10_O_7_.xH_2_O, 302.24 kDa, anhydrous basis, purity ≥95%; obtained from Sigma-Aldrich) dissolved in dimethyl sulfoxide (DMSO; Sigma-Aldrich) was added to the culture medium to yield a final concentration of 100 μM. Cells were monitored for ~170 h at 37˚C in a 5% CO_2_ atmosphere, with one change of freshly-prepared 100 μM quercetin and medium at 72-h post-seeding. Dynamic real-time monitoring of the growth inhibition pattern was determined by the impedance-based xCELLigence system (Roche Diagnostics GmbH, Mannheim, Germany). The cell index was derived from measured cell-electrode impedance which correlates with number of cells, viability and/or cytotoxicity. Control cultures received DMSO alone. The final concentration of DMSO in the cultures was ≤0.5%.

### Generation of xenografts

Stable HK1 transfectants were resuspended in 1:1 serum-free medium and BD Matrigel Basement Membrane Matrix at a concentration of 1.0×10^6^ cells. Nude mice (4–6 weeks-old) were subcutaneously injected on the upper right flank under mild anaesthesia. Living conditions of the animals were monitored routinely. Following 28 days of inoculation, the animals were euthanized and tumours were excised. A section of the excised tumour was snap frozen in liquid nitrogen and the remainder fixed in 10% formalin and paraffin-embedded (FFPE). All procedures were conducted in accordance to the appropriate ethics guidelines of the Ministry of Health (MOH; Malaysia) and approved by the Animal Care and Use Committee (MOH).

### EBV ISH

FFPE (4 μm) tissue sections were placed on silanized glass slides. ISH was performed using the Epstein-Barr Virus Fluorescein-conjugated Probe for ISH kit (Novocastra, Newcastle-upon-Tyne, UK), according to the manufacturer’s instructions. Sections from the known EBV-positive xenograft, C15 (16), maintained in nude mice, were used as positive controls. The negative control was performed by substituting the EBV probe with a control probe consisting of a fluorescein-labelled random oligonucleotide cocktail.

### Statistical analysis

Calculations were performed using SPSS 13.0 statistical software for Windows (SPSS Inc., Chicago, IL, USA). Differences between mean values were evaluated with the Student’s t-test or one-way analysis of variance and Tukey’s post hoc analysis. P<0.05 was considered to indicate a statistically significant difference.

## Results

### Establishment of vector- and EBER-transfected HK1 stable cell lines

**Figure 1:**
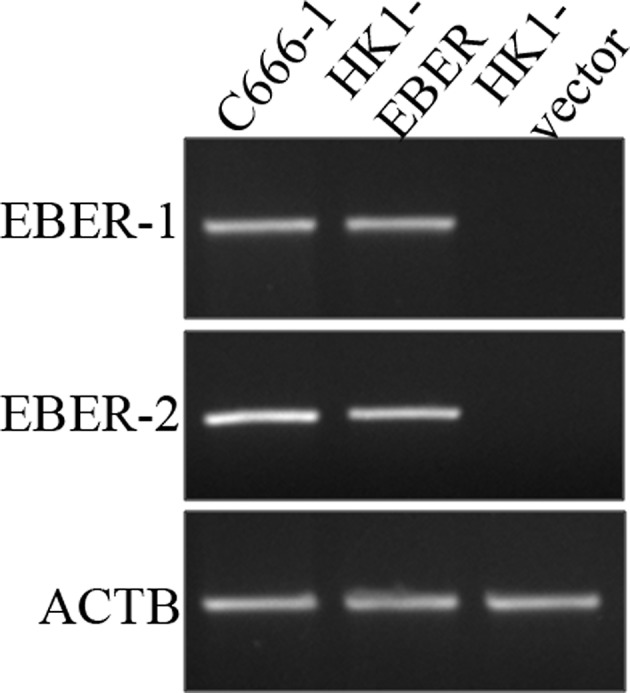
Establishment of HK1 stable cell line harbouring EBER plasmid or control vector. HK1, an EBV-negative NPC cell line, was transfected with EBER plasmid or empty vector and expression of EBER-1 and -2 was determined by RT-PCR. C666-1, an EBV-positive NPC cell line, was used as a positive control, whereas ACTB served as the housekeeping gene. EBV, Epstein-Barr virus; EBERs, EBV-encoded RNAs; RT-PCR, reverse transcription polymerase chain reaction; NPC, nasopharyngeal carcinoma; ACTB, β-actin.

To investigate the role of EBERs in NPC, EBER-plasmid or vector alone (control) was introduced via liposome-mediated transfection in NPC HK1 cells. Stable transfectants were generated and examined for the presence of EBERs by RT-PCR. The cell line generated by this approach (designated as HK1-EBER) successfully expressed EBER-1 and -2, while no EBERs were detected in HK1 cells transfected with vector plasmid (HK1-vector), which served as the control (Fig. 1).

### Effect of EBERs on cell proliferation and morphology

**Figure 2:**
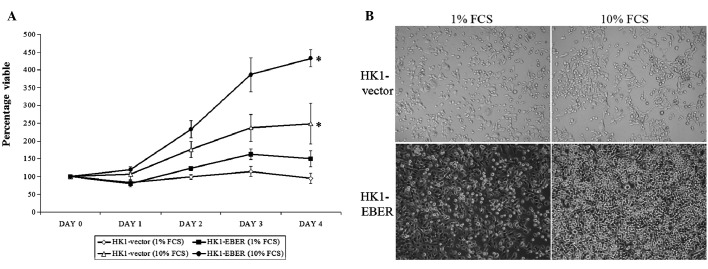
Effect of EBER expression on cell proliferation. (A) Growth curves depicting proliferation of HK1-vector or -EBER in 1 and 10% FCS, determined by the MTS assay. HK1-EBER exhibited an increased growth rate compared with the HK1-vector. Three independent experiments involving four replicates/time-point were conducted. The growth curves were plotted as % of viable cells vs. day 0 and represent the average of one independent experiment, ^*^P<0.05. (B) Representative photomicrographs of the stable transfectants in culture medium supplemented with 1 or 10% FCS (magnification, ×100). HK1-EBER reached confluence faster than the control. Similar results were obtained in two additional experiments. EBERs, Epstein-Barr virus-encoded RNAs; FCS, foetal calf serum.

Stable cell lines were assessed for growth. On Days 1–4, HK1-EBER exhibited a higher proliferation rate compared with the control. On Day 4, the proliferation capacity of HK1-EBER in full medium (10% FCS) was observed as significantly greater than the control (Fig. 2A). In low serum, lack of growth factors affected the growth of HK1-EBER and the higher proliferation rate observed in full medium was not found to be statistically significant compared with HK1-vector under these conditions. Growth patterns and morphological changes in culture medium supplemented with 1 and 10% FCS were observed using light microscopy (Fig. 2B). HK1-vector and -EBER cells were observed under light microscopy as adherent with similar morphologies. In addition, microscopic examination clearly showed that HK1-EBER cells reached confluence more rapidly than the control under normal growth conditions. These observations were consistent with growth patterns obtained by MTS assay (Fig. 2A).

### EBERs do not alter sensitivity of NPC cells to cisplatin

**Figure 3:**
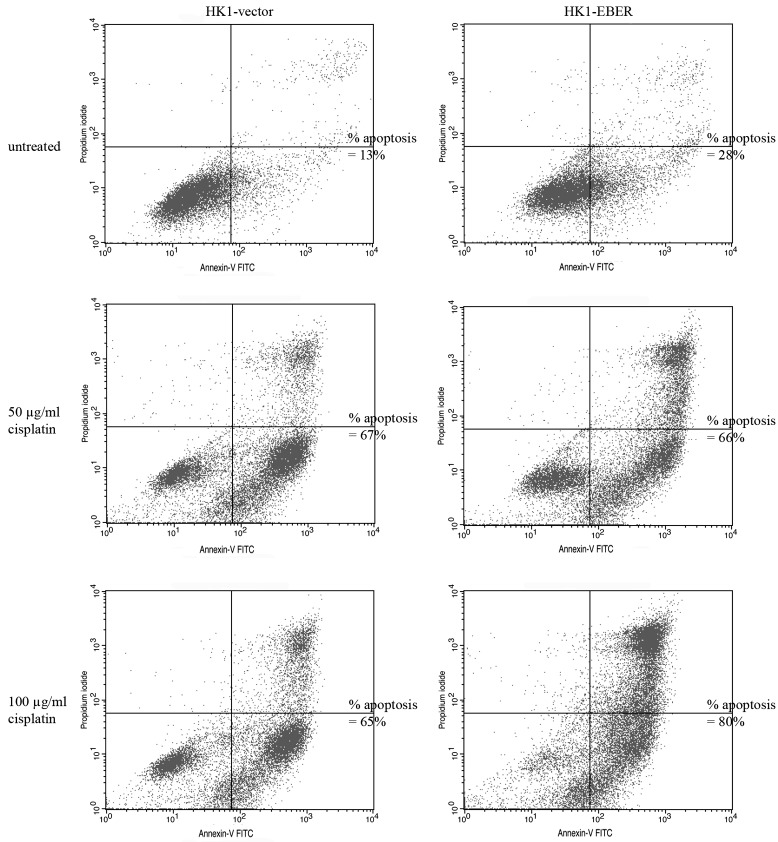
Flow cytometry analysis using Annexin V-FITC/PI double staining showing apoptosis. Cells were exposed to cisplatin 24 h prior to analysis. The lower right and upper right quadrants are cells undergoing apoptosis. Images shown are representative of three independent experiments. Average percentage of total apoptotic cells is indicated.

To determine whether EBERs confer resistance to apoptosis-induction, stable cell lines were cultured in the presence of 50–100 μg/ml cisplatin, a drug commonly used for treatment of NPC. Apoptotic cells were identified by flow cytometry. Cisplatin clearly induced apoptosis in HK1-vector and -EBER stable transfectants (Fig. 3). Treatment with 100 μg/ml cisplatin for 24 h caused ~50% cell death following correction of background apoptosis (Fig. 3). Under these conditions, no difference in the percentage of dead cells between the two stable transfectants was found.

### EBERs do not alter migration and invasion of NPC cells

**Figure 4:**
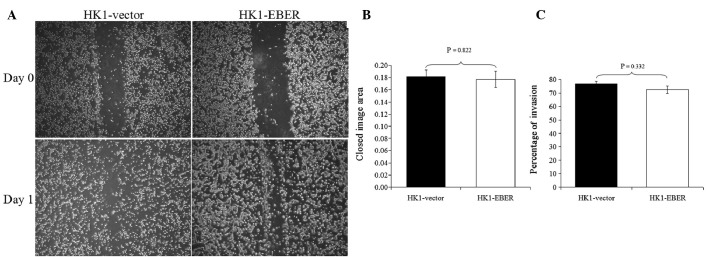
Scratch-wound assay and *in vitro* FluoroBlok tumour invasion assay. (A) A line scratch was made and photomicrographs were captured at days 0 and 1 (magnification, ×50). (B) Using the TScratch program, a software tool for automated analysis of wound healing assays, the open image area was calculated; from which the closed imge area (reflecting the degree of migration or would healing) was derived. No significant difference in migration ability was identified between the two stable cell lines. (C) Quantification of fluorescence emitted by cells that have invaded to the underside of the FluoroBlok membrane was detected with a plate reader and the % of cell invasion was calculated. No significant difference in invasiveness was observed between the cell lines. EBERs, Epstein-Barr virus-encoded RNAs.

To determine whether EBERs affect cell migration and invasion, HK1-EBER and -vector were subjected to scratch-wound (17) and FluoroBlok invasion assays. HK1-EBER and -vector cells exhibited similar migration and invasion patterns (Fig. 4). EBER expression did not affect migration and invasion in HK1 NPC cells.

### Deregulation of the cellular lipid metabolic pathway

**Figure 5:**
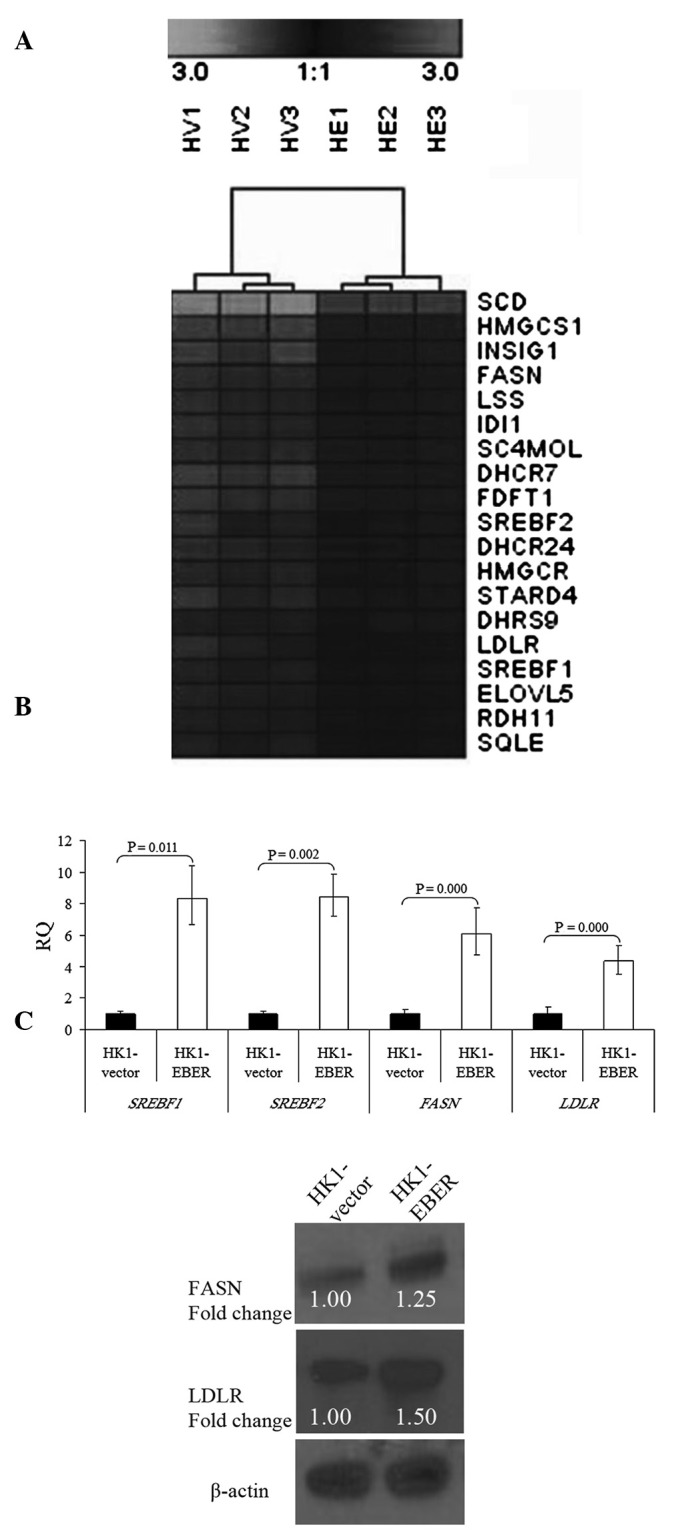
Hierarchical clustering of transcripts and validation of gene expression. (A) Selected gene expression clusters of functionally annotated gene sets representing the cellular lipid metabolic process. Varying shades represent downregulation and upregulation (P<0.05). HV, HK1-vector; HE, HK1-EBER. (B) qRT-PCR of *SREBF1* and *2*, *FASN* and *LDLR* in HK1-EBER and HK1-vector. mRNA levels of *SREBF1* and *2*, *FASN* and *LDLR* were found to be significantly increased in HK1-EBER (P<0.05). ACTB and PPIA were used as multiple controls for normalization. Relative expression levels were calculated by 2^-ΔΔCt^ and reported as RQ. (C) Overexpression of FASN and LDLR in HK1-EBER demonstrated by western blot analysis. ACTB was used as the loading control. Similar results were obtained in two subsequent independent experiments. EBERs, Epstein-Barr virus-encoded RNAs; qRT-PCR, quantitative reverse transcription polymerase chain reaction; SREBF, sterol regulatory element binding protein; FASN, fatty acid synthase; LDLR, low-density lipoprotein receptor; ACTB, β-actin; PPIA, peptidylprolyl isomerise A; RQ, relative quantification.

To determine transcriptome-wide gene expression profiles associated with EBERs, we performed microarray analysis in RNA extracted from HK1-EBER and -vector cells. Following stable transfection, RNA was prepared from the cell lines and hybridized to oligonucleotide microarray chips. Significant differential gene expression was defined using the unpaired t-test (P<0.05; threshold, ≥2-fold change). Under these criteria, a total of 54 gene transcripts were observed as significantly upregulated and 155 were significantly downregulated by the EBERs. GO analysis indicated that 19 genes upregulated by EBERs were significantly overexpressed in the cellular lipid metabolic process (Fig. 5A and Table I). Hierarchical clustering was performed and a heat map was generated using Genesis IGB-TUG Software (18). *FASN*, *LDLR* and *SREBF1* and *2* were selected from our gene chip data for further validation. Consistent with microarray findings, qRT-PCR confirmed significant overexpression of *FASN*, *LDLR* and *SREBF1* and *2* in HK1-EBER cells compared with control (Fig. 5B). FASN and LDLR protein levels were identified by western blot analysis as markedly elevated in HK1-EBER (Fig. 5C). Together with microarray and qRT-PCR results, these observations indicate upregulation of the cellular lipid metabolic process in HK1-EBER cells.

### The role of LDL receptor in NPC

**Figure 6:**
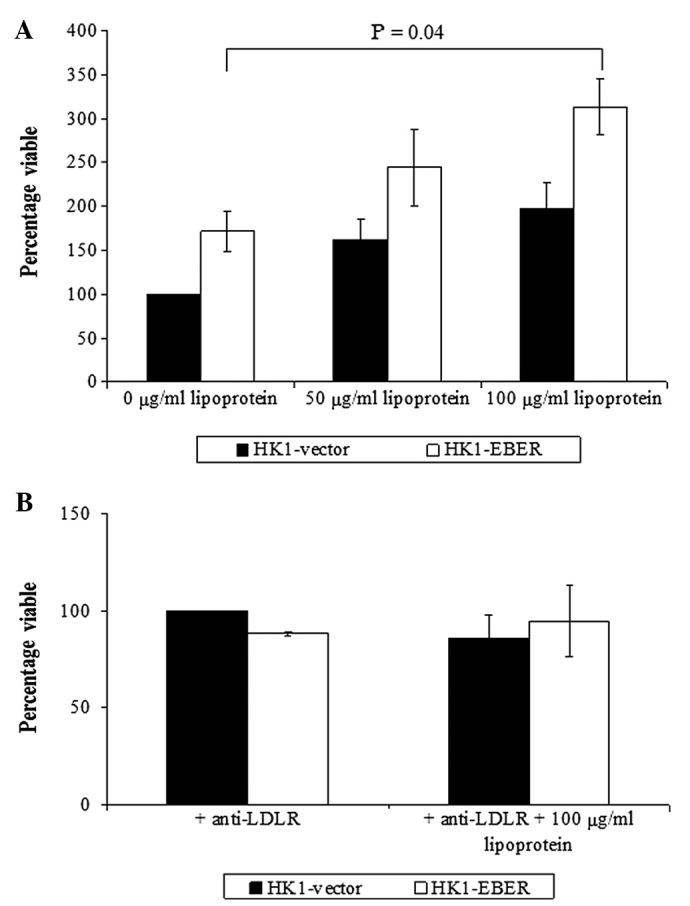
(A) Effects on proliferation by addition of lipoprotein. HK1-EBER and -vector cells cultured in serum-free culture medium only or culture medium and lipoprotein were subjected to MTS assay. Cells exhibited lipoprotein-dependent proliferation. Bars are the average of three independent experiments and Y-error bars represent SEM. P<0.05, % of viable cells in lipoprotein-supplemented culture medium vs. culture medium without lipoprotein. (B) Effect of blockade of LDLR-ligand interaction by anti-human LDLR antibody on LDL-induced proliferation. Cell proliferation was not induced by the addition of lipoprotein following inhibition of LDLR-ligand interaction. P>0.05, cells with unsupplemented vs. lipoprotein-supplemented culture medium. Bars are the average of three independent experiments and Y-error bars represent SEM. EBERs, Epstein-Barr virus-encoded RNAs; LDLR, low-density lipoprotein receptor; SEM, standard error of mean.

As LDLR was elevated in HK1-EBER, we investigated whether supplementation of LDL stimulates NPC cell growth. Although stimulation of cell growth was observed in HK1-EBER and -vector, significantly increased growth was identified in HK1-EBER cells treated with 100 μg/ml LDL only (Fig. 6A). Inhibition of the LDL receptor with anti-human LDLR antibody blocked LDL-induced cell proliferation (Fig. 6B).

### Effects of quercetin on HK1-vector and -EBER cells

**Figure 7:**
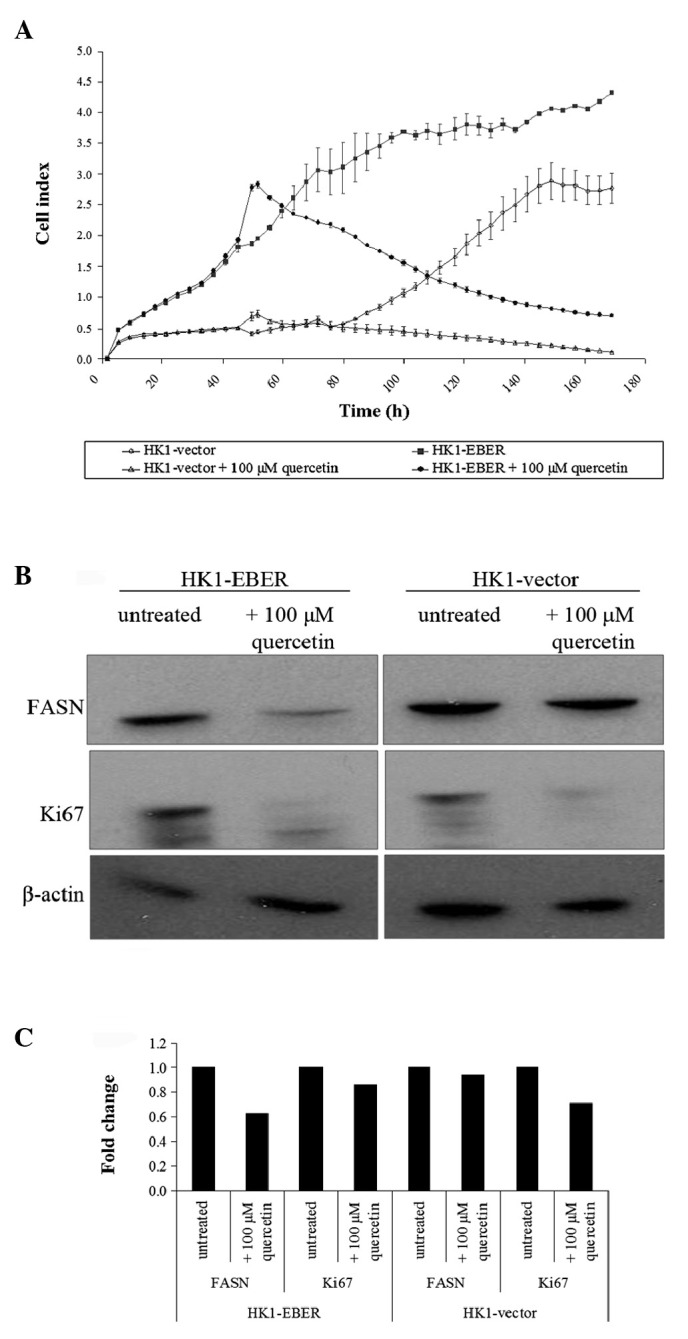
Effects of quercetin on NPC cells. (A) Real-time analysis of NPC cell proliferation in the presence and absence of quercetin. Cell index data demonstrate that in the absence of quercetin, HK1-EBER exhibited an increased growth rate compared with the vector control. Quercetin-treatment inhibits HK1-EBER and -vector cell proliferation. The graph is representative of triplicate experiments. (B) Immunoblot for FASN and Ki67 24 h following quercetin treatment. NPC cells treated with quercetin expressed lower amount of FASN and Ki67 compared with untreated cells. ACTB served as a loading control. The experiment was repeated twice and consistent results were obtained. A representative result is presented. (C) Bar graphs show densitometric analysis of protein levels relative to untreated cells. NPC, nasopharyngeal carcinoma; EBERs, Epstein-Barr virus-encoded RNAs; FASN, fatty acid synthase; ACTB, β-actin.

Quercetin was previously reported to inhibit FASN (19). To determine the effects of quercetin on EBER-expressing cells, HK1 stable transfectants treated (or untreated) with quercetin were tested by RT xCELLigence cell proliferation assay. The cell index demonstrated represents growth over time. When untreated, HK1-EBER cells proliferated faster than control (Fig. 7A), consistent with the MTS assay performed (Fig. 2A). Quercetin treatment inhibited HK1-EBER and -vector cell proliferation (Fig. 7A), consistent with a previous report that quercetin inhibited NPC cells (20).

Using western blot analysis, we demonstrated that quercetin-treatment resulted in the downregulation of FASN and Ki67 proliferation antigen expression (Fig. 7B and C). This observation was consistent with Fig. 7A which revealed that cell growth halted in the presence of quercetin. The connection between FASN expression and the Ki67 marker of proliferation indicates that FASN may be associated with cell proliferation.

### EBER-1 and -2, FASN, LDLR and SREBF1 and 2 expression in xenografts

**Figure 8:**
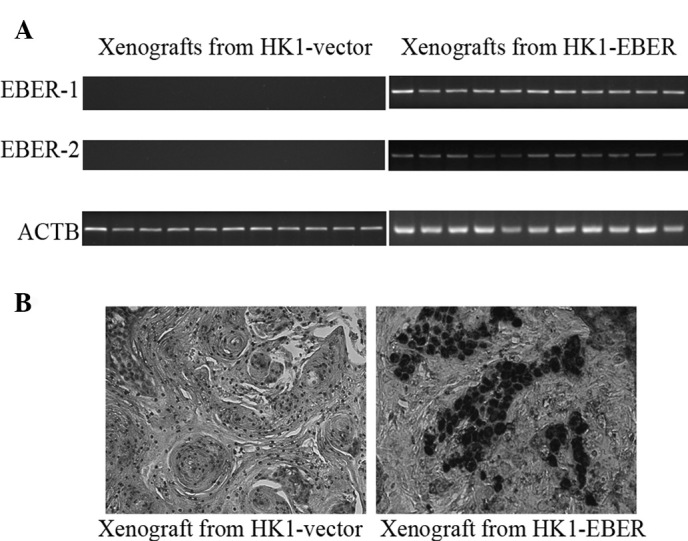
Presence of EBERs in xenografts determined by: (A) RT-PCR and (B) EBV ISH. (A) RNA was isolated from excised xenografts and subjected to RT-PCR for EBER-1 and -2. All 11 HK1-EBER xenografts demonstrated marked presence of EBER-1 and -2. (B) Intense black staining on FFPE sections indicates marked nuclear hybridisation of the EBER probe which occurred only in xenografts developed from HK1-EBER. Results are consistent with RT-PCR results. EBV, Epstein-Barr virus; EBERs, EBV-encoded RNAs; RT-PCR, reverse transcription polymerase chain reaction; ISH, *in situ* hybridisation; FFPE, formalin-fixed, paraffin-embedded.

**Figure 9:**
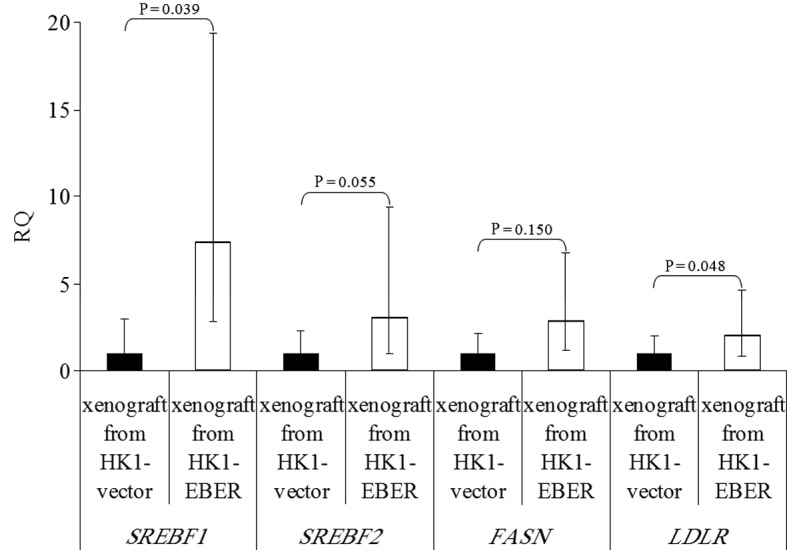
Expression of *SREBF1* and *2*, *FASN* and *LDLR* in xenografts formed from HK1-EBER and the control, quantified by qRT-PCR. The results are expressed as RQ to the control xenografts following normalization to the endogenous control ACTB. The trend in differential gene expression *in vivo* was consistent with that observed *in vitro*. P>0.05, HK1-EBER overexpression of *SREBF2* and *FASN* vs. control. P≤0.05, HK1-EBER overexpression of *SREBF1* and *LDLR* vs. control. Limited significance may be explained by a large variation in expression levels. SREBF, sterol regulatory element binding protein; FASN, fatty acid synthase; LDLR, low-density lipoprotein receptor; EBERs, Epstein-Barr virus-encoded RNAs; qRT-PCR, quantitative reverse transcription polymerase chain reaction; RQ, relative quantification; ACTB, β-actin.

HK1-EBER-transfected xenografts and controls (11 each) were tested for expression of EBERs by RT-PCR and EBV ISH. All HK1-EBER-transfected xenografts were positive for EBERs and all controls were negative (Fig. 8A and B). All 11 pairs of xenografts were then analysed by real-time qPCR for *FASN*, *LDLR*, *SREBF1* and *2* expression. EBER-positive xenografts had variable, although a generally higher expression of genes associated with the cellular lipid metabolic process (Fig. 9), in agreement with *in vitro* data (Fig. 5B).

## Discussion

A number of studies have reported an association between malignancies and metabolic syndromes, including atherosclerosis, diabetes and obesity. Hirsch *et al* reported common gene networks linking cancer with lipid metabolism. The authors of that study noted that multiple genes involved in lipid metabolism, cholesterol biosynthesis and atherosclerosis, including OLR1 (oxidized LDL receptor 1), SCD1, SREBP1, SNAP23 and VAMP4 were deregulated in cell transformation. OLR1, GLRX and SNAP23 were overexpressed in human prostate and breast cancer tissues and high expression levels of these molecules are associated with aggressive phenotype and metastatic stage (21).

The present study demonstrates that EBERs deregulate the cellular lipid metabolic process, contributing to elevation of the FASN enzyme and LDLR. EBERs increased proliferation of NPC cells. In addition, NPC cells demonstrated LDL-dependent cell proliferation. The ability of a polyphenolic flavonoid, quercetin, to inhibit FASN and cell proliferation was also revealed in NPC cells.

LDLR is a cell surface glycoprotein that mediates the uptake of LDL via endocytosis to be delivered to extrahepatic tissues for membrane synthesis (22). In specific cancer cells, including acute myelogenous leukemia (23), prostate (24,25) and colorectal cancer (26), feedback regulation of LDLR is lacking, leading to excess energy sources and subsequent stimulation of uncontrolled growth. Cell growth, differentiation and neoplastic transformation alter LDLR levels (27). Increased LDLR activity of tumour cells may occur to facilitate increased LDL uptake to satisfy the high cholesterol demand for cell growth or mechanisms associated with cell transformation (28). Consistent with the hypothesis that LDLR promotes proliferation, we demonstrated that HK1-EBER, which exhibited accelerated cell proliferation (Fig. 2), also expressed higher levels of LDLR (Fig. 5B and C).

Lum *et al* studied the effect of LDL on growth and gene regulation in DiFi colorectal cancer cells (26). When cultured in lipoprotein-deficient serum, the growth of DiFi cells was reduced compared with culture with LDL. In the presence of excess LDL, uptake of LDL continued indicating a lack of normal feedback regulation. However, when LDLR blocking antibody was added to growing cells, growth was observed to be significantly reduced. As demonstrated in Fig. 6A, when exogenous LDL was added to serum-free culture medium, the growth of HK1-EBER was significantly increased. Serum-free culture medium was necessary to study the effect of exogenous LDL supplementation as FCS contains lipoproteins, insulin and additional growth factors that affect LDLR activity (29). NPC cells were able to uptake LDL via endocytosis and utilised LDL to boost growth in the absence of FCS. However, cell proliferation was not induced by the addition of lipoprotein following inhibition of LDLR-ligand interaction. We hypothesise that this was due to blocked receptors being unable to uptake lipoprotein for proliferation. These results confirm LDL-dependent cell proliferation and indicate that LDLR may be crucial for proliferation of NPC cells.

SREBP1 and 2 are an important family of transcription factors for cholesterol and fatty acid synthesis. SREBP1 is encoded by the *SREBF1* gene and is involved in fatty acid metabolism. SREBP2 is encoded by the *SREBF2* gene and is involved in cholesterol synthesis. SREBPs activate >30 genes, including FASN, LDLR, 3-hydroxy-3-methyl-glutaryl (HMG)-CoA reductase and synthase and stearoyl-CoA desaturase (30,31), all of which were upregulated by EBERs in the current study (Fig. 5A). Previously, EBERs were reported to induce IGF-1 in the EBV-positive C666-1 NPC cell line and in EBER-transfected NPC-derived EBV-negative CNE1 and HONE1 cell lines (10), as well as in EBER-transfected gastric carcinoma NU-GC-3 cells (9). SREBP1 is associated with the insulin and IGF-1 signal transduction pathway, leading to activation of the *LDLR* gene promoter (32). An isoform of SREBP1, SREBP1c, was previously identified as a major mediator of insulin action on the hepatic expression of lipogenesis-related genes (33). Streicher *et al* studied the mechanisms that stimulate the promoter activity of *LDLR* in HepG2 cells (32). Those authors observed that hormones, including insulin and IGF-1 increase *LDLR* mRNA concentration in the presence of repressive concentrations of LDL. In a study by LaVoie *et al*, 100 ng/ml IGF-1 significantly stimulated *LDLR* expression in serum-free cultures of swine granulosa cells. IGF-1 and follicle stimulating hormone increased LDLR binding and internalization and utilisation of LDL (34). Therefore, upregulation of lipid metabolism genes is consistent with the upregulation of IGF-1 by EBERs.

In normal cells, FASN produces a 16-carbon saturated fatty acid at the expense of nicotinamide adenine dinucleotide phosphate (35). Human cancer cells have elevated levels of FASN and undergo exacerbated endogenous fatty acid synthesis independent of regulatory signals that downregulate fatty acid synthesis in normal cells (36). Increased *de novo* fatty acid synthesis has been recognised as a hallmark of cancer (36). In cancer cells, endogenously synthesized fatty acids are stored as phospholipids (compared with triglycerides in normal cells) which are incorporated into biological membranes of proliferating tumour cells (37). As fatty acid synthesis is necessary to maintain a constant supply of lipids and lipid precursors for membrane production in a highly-proliferating population (36), it is assumed that increased fatty acid synthesis aids tumour growth. Elevated FASN was identified in human breast, bladder, colon, head and neck, endometrium, lung, prostate, oesophagus, ovary, stomach, tongue and thyroid cancers (35,37).

Quercetin (3,3′,4′,5,7-pentahydroxyflavone) is a polyphenolic flavonoid widely distributed in fruits and vegetables. Previously, quercetin was identified to inhibit enzymatic activity of FASN and arrest cell growth (19). We recently demonstrated that quercetin inhibited cell proliferation and decreased the expression of FASN in NPC cells (20). In addition, quercetin was demonstrated to effectively inhibit growth in EBV-negative NPC HEN1 (38), human gastric cancer HGC-27 (39) and human leukaemia T-cells (40). Naturally occurring polyphenols, including the flavonoids luteolin, quercetin and kaempferol, were the most effective FASN inhibitors in breast and prostate cancer cells and were associated with cell growth arrest and apoptosis induction, indicating that flavonoids may exert anti-carcinogenic effects via FASN inhibition (41). In the present study, FASN was inhibited by 100 μM quercetin treatment while tumour cell proliferation ceased and the bands obtained in western blot analysis were less intense for the Ki67 proliferation antigen in response to quercetin (Fig. 7), demonstrating that lipid metabolism may be associated with cell proliferation. FASN expression has been reported to correlate with Ki67 labelling index in human endometrial carcinomas (42). This close and direct link between FASN expression and Ki67 marker of proliferation indicates that FASN is associated with cell proliferation. According to Kuhajda (35,37), a rapid decline occurs in fatty acid synthesis following FASN inhibition. In addition, cell cycle arrest was induced, leading to a decrease in tumour cell proliferation and ultimately apoptosis, indicating that cancer cells are dependent on fatty acid synthesis for survival. Inhibition of FASN may be responsible for cancer cell death as FASN inhibition introduces changes in the synthesis of membrane phospholipids (36). Findings of the present study demontstrated that quercetin was able to inhibit cell proliferation and may present a means to block uncontrolled growth of cancer cells expressing increased levels of fatty acid synthase. These preliminary results provide additional evidence for the potential of quercetin in NPC.

The data generated in this study suggest that EBERs may contribute to the deregulation of the cellular lipid metabolic process pathway in NPC cells. Aggressive growth was associated with elevated FASN and LDLR expression induced by EBERs in the cell lines tested. Results demonstrate that the cells exhibited LDL-dependent cell proliferation. In addition, cell growth may be inhibited by quercetin treatment.

The present study was primarily performed in EBV-negative HK1 NPC cells which were stably transfected with EBERs. The aim of this study was to determine whether EBERs alter the biology of a pre-existing cancer cells. While lipid metabolism was reported to share specific common gene signatures with cell transformation, our data were performed on pre-existing cancer cells and analysed lipid metabolism genes which had been previously associated with prognosis (36,37), indicating that lipid metabolism may share specific common features with cancer progression as well.

In the present study, we generated stable transfectants expressing EBERs, enabling multiple assays to be performed in similar cells. However, the use of stable transfectants requires selection of clones by antibiotics (G418). It is plausible that the cells which were selected had incidental pre-existing difference in biological properties not associated with EBERs. Pooling of resistant clones was performed to minimize the effects of selected spurious clones. In this preliminary study, we did not distinguish whether the altered biology of cells was due to direct or indirect effects of EBERs. However, as discussed, the effects of EBERs on the deregulation of lipid metabolism genes are likely to be indirect.

Lipid metabolism genes may be affected by several pathways. There is wide variability in the expression levels of the genes found in xenografts generated from these cells, although the trend is consistent with the gene expression studies obtained from *in vitro* experiments. Gene expression levels in xenografts may be altered by additional factors, including tumour microenvironmental factors.

We hypothesise that the effect of EBERs was mediated through LDLR and FASN as those genes were confirmed to be upregulated at the transcript and protein levels. It is plausible that the increased effect of quercetin observed in EBER-positive cells was simply due to a higher proliferative rate than EBER-negative cells. Nevertheless, it is noteworthy that LDL and quercetin had an effect on NPC cells and that this effect was more marked in EBER-positive cells, which were of higher proliferative rate.

Further validation of the association between EBERs and lipid metabolism pathway in additional cell types is important. In addition, the effect of knocking down EBERs in EBV-positive cells may provide useful information. Understanding the effects of deregulation of lipid metabolism on cancer progression is likely to be of significant interest as manipulation of these pathways may lead to new approaches of therapeutic targeting in a number of different types of cancer, including NPC.

## Figures and Tables

**Table I:** GO classification of upregulated genes in HK1-EBER.

GO (biological process)	No. of genes upregulated	P-value
Cellular lipid metabolic	19	1.09E-15
Steroid metabolic	15	1.48E-15
Lipid metabolic	19	2.34E-14
Sterol metabolic	12	2.34E-14
Lipid biosynthetic	13	2.42E-11
Steroid biosynthetic	10	2.64E-11
Cholesterol metabolic	10	3.97E-11
Sterol biosynthetic	8	4.54E-11
Cellular biosynthetic	18	1.19E-09
Alcohol metabolic	12	3.08E-08
Cholesterol biosynthetic	6	1.16E-07
Isoprenoid biosynthetic	4	8.11E-04
Biosynthetic	18	0.004

Significance analysis conducted using the unpaired t-test at P<0.05 and fold-change ≥2.0. GO, Gene Ontology; EBER, Epstein-Barr virus-encoded RNAs.
